# WEE1 kinase is a therapeutic vulnerability in CIC-DUX4 undifferentiated sarcoma

**DOI:** 10.1172/jci.insight.152293

**Published:** 2022-03-22

**Authors:** Rovingaile Kriska M. Ponce, Nicholas J. Thomas, Nam Q. Bui, Tadashi Kondo, Ross A. Okimoto

**Affiliations:** 1 Department of Medicine, UCSF, San Francisco, California, USA.; 2 Department of Medicine, Stanford University School of Medicine, Stanford, California, USA.; 3 Division of Rare Cancer Research, National Cancer Center Research Institute, Tokyo, Japan.; 4 Helen Diller Family Comprehensive Cancer Center, UCSF, San Francisco, California, USA.

**Keywords:** Oncology, Cancer

## Abstract

CIC-DUX4 rearrangements define an aggressive and chemotherapy-insensitive subset of undifferentiated sarcomas. The CIC-DUX4 fusion drives oncogenesis through direct transcriptional upregulation of cell cycle and DNA replication genes. Notably, CIC-DUX4–mediated *CCNE1* upregulation compromises the G_1_/S transition to confer a dependence on the G_2_/M cell cycle checkpoint. Through an integrative transcriptional and kinase activity screen using patient-derived specimens, we now show that CIC-DUX4 sarcomas depend on the G_2_/M checkpoint regulator WEE1 as part of an adaptive survival mechanism. Specifically, CIC-DUX4 sarcomas depended on WEE1 activity to limit DNA damage and unscheduled mitotic entry. Consequently, genetic or pharmacologic WEE1 inhibition in vitro and in vivo led to rapid DNA damage–associated apoptotic induction of patient-derived CIC-DUX4 sarcomas. Thus, we identified WEE1 as a vulnerability targetable by therapeutic intervention in CIC-DUX4 sarcomas.

## Graphical abstract

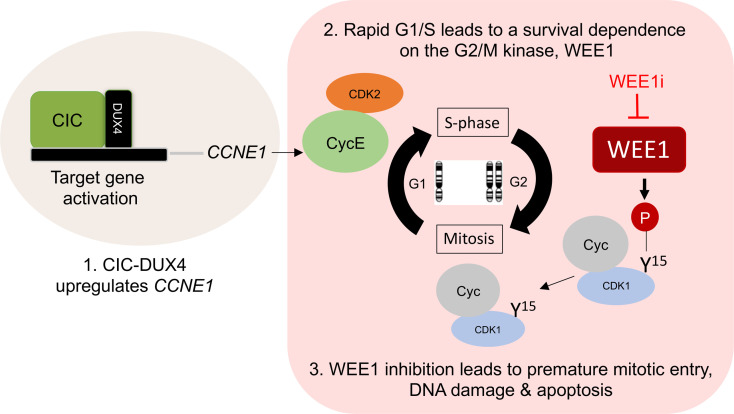

## Introduction

Chromosomal rearrangements that create transcription factor (TF) fusion oncoproteins are attractive cancer-specific therapeutic targets (1). For example, mechanistic studies that provide insight into fusion oncoprotein stability have led to the development of precision medicine–based therapies that directly degrade these driver oncoproteins (1–3). Beyond direct degradation, an alternative strategy to overcome TF fusion dependence in cancer is identification of key transcriptional targets directly controlled by TF fusions to drive malignant progression. To this end, we recently observed that CIC-DUX4 sarcoma is molecularly dependent on the CCNE/CDK2 complex: CIC-DUX4 acquires neomorphic function as a transcriptional activator to upregulate *CCNE1,* driving sarcoma growth and survival (4). Therapeutic intervention with CDK2 inhibitors leads to induction of apoptosis in models using patient-derived CIC-DUX4 samples (4). These findings provided a mechanism-based therapeutic strategy to limit CIC-DUX4 sarcoma progression, which remains an aggressive and lethal disease.

In other cancers, increased expression of *CCNE1* through transcriptional upregulation or amplification often leads to a deficient G_1_/S checkpoint, thus enhancing DNA replication stress and genomic instability (5–11). Cancer cells adapt to these high-replication-stress states through an increased dependence on cell replication checkpoints that enable accurate DNA repair, scheduled mitotic entry, and survival (8, 12). When these key checkpoints are compromised, cancer cells undergo replication stress–driven mitotic catastrophe and death (13, 14). One critical cell cycle checkpoint regulator is the WEE1 kinase, which modulates CDK1 and CDK2 activity through direct inhibitory phosphorylation (15, 16). Functionally, phosphorylation at tyrosine 15 (Y15) on CDK1 by WEE1 delays progression at the G_2_/M checkpoint (15, 17), restricting premature entry into mitosis. Thus, as part of a mechanism of adaptation to CCNE1-associated replication stress, WEE1 can enhance tumor cell survival. Through an integrative transcriptional and functional approach, we investigated how CIC-DUX4 sarcomas survive in a CCNE1-mediated high-replication-stress state through increased dependence on the WEE1 kinase. Moreover, we demonstrate that WEE1 is a therapeutic vulnerability in CIC-DUX4 sarcomas that can be targeted with clinically advanced WEE1 inhibitors.

## Results

**Figure 1:**
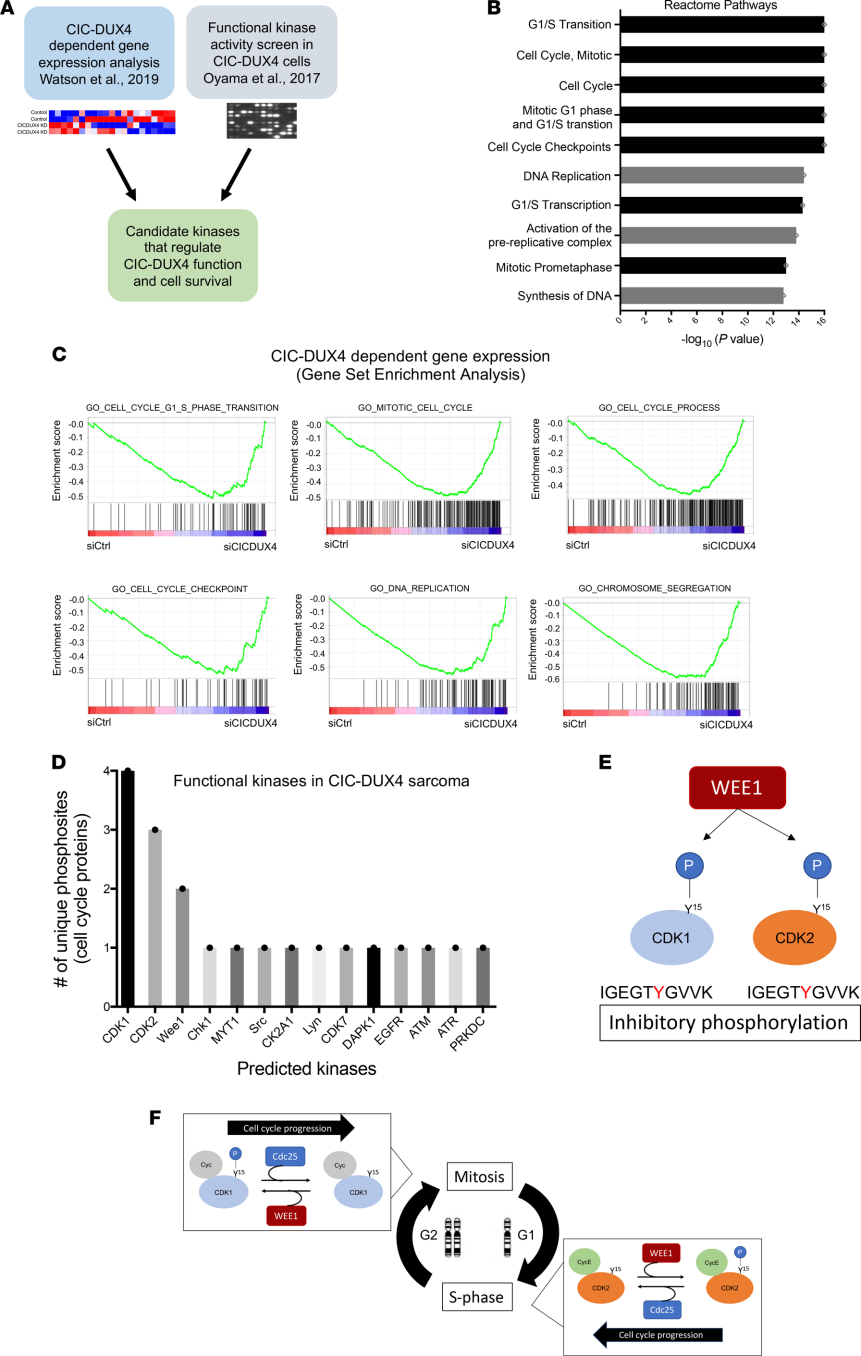
Integrative transcriptomic and kinase activity screen identifies WEE1 as a target in CIC-DUX4 sarcoma. (**A**) Approach to identifying candidate kinases that regulate CIC-DUX4 sarcoma survival. Watson et al., 2019: ref. 19; Oyama et al., 2017: ref. 22. (**B**) Reactome pathway analysis identifies CIC-DUX4–dependent cell cycle and DNA replication pathways. (**C**) GSEA reveals cell cycle, DNA replication, and chromosome segregation at CIC-DUX4 targets. (**D**) PamGene array identifies functional kinases that regulate cell cycle and DNA replication in CIC-DUX4 cells. (**E**) Model of WEE1 inhibitory kinase motifs in CDK1 and CDK2. (**F**) Schematic of WEE1-regulated cell cycle control.

**Figure 2:**
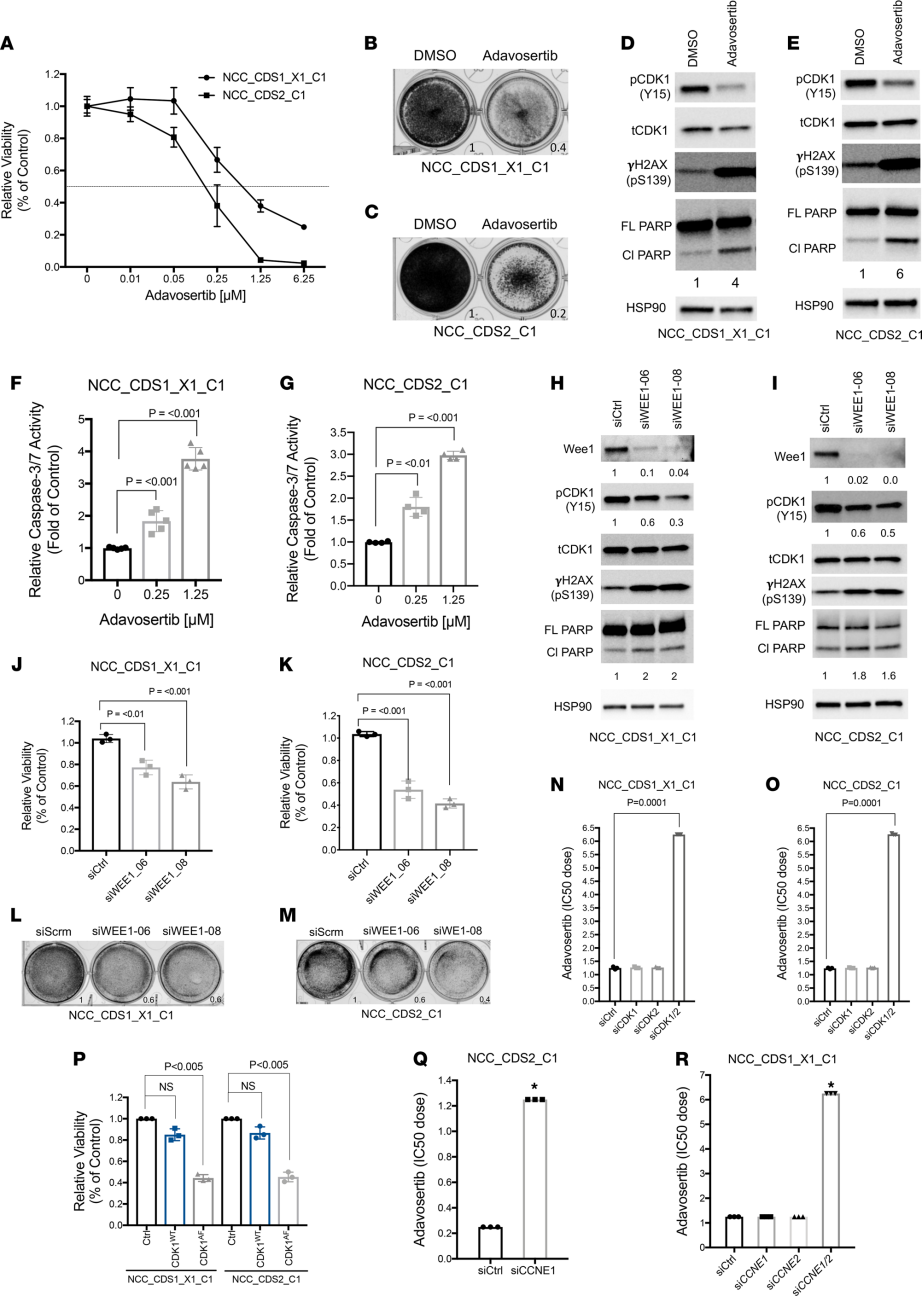
CIC-DUX4 sarcomas depend on WEE1 for survival. (**A**) CTG viability assay of NCC_CDS1_X1_C1 and NCC_CDS2_C1 cells treated with adavosertib. Error bars represent SEM; performed in duplicate. Crystal violet assay of NCC_CDS1_X1_C1 (**B**) and NCC_CDS2_C1 (**C**) cells comparing adavosertib (IC_50_ dose) and DMSO control. (**D**) Immunoblot analysis of NCC_CDS1_X1_C1 cells treated with adavosertib or DMSO control. Representative figure; performed in duplicate. Lysates were run on parallel gels. FL, full-length; Cl, cleaved. (**E**) Immunoblot analysis of NCC_CDS2_C1 cells treated with adavosertib or DMSO control. Representative figure; performed in duplicate. (**F** and **G**) Relative caspase-3/7 activity in NCC_CDS1_X1_C1 and NCC_CDS2_C1 cells treated with adavosertib versus control. One-way ANOVA; performed in triplicate. (**H** and **I**) Immunoblot analysis of NCC_CDS1_X1_C1 and NCC_CDS2_C1 cells expressing 2 independent WEE1 siRNAs. Representative figure; performed in duplicate. (**J** and **K**) CTG viability assay comparing 2 independent WEE1 siRNAs with scramble control in NCC_CDS1_X1_C1 and NCC_CDS2_C1 cells. One-way ANOVA; performed in triplicate. (**L** and **M**) Viability assay comparing 2 independent WEE1 siRNAs with scramble control (siScrm) in NCC_CDS1_X1_C1 and NCC_CDS2_C1 cells. One-way ANOVA; performed in duplicate. (**N** and **O**) Adavosertib IC_50_ dose in NCC_CDS1_X1_C1 and NCC_CDS2_C1 cells expressing siCDK1, siCDK2, or siCDK1 and siCDK2. One-way ANOVA; performed in triplicate. (**P**) Relative viability comparing NCC_CDS1_X1_C1 and NCC_CDS2_C1 cells expressing CDK1^WT^ or CDK1^AF^ versus vector control. One-way ANOVA. Error bars represent SEM; performed in triplicate. (**Q**) Adavosertib IC_50_ dose in NCC_CDS2_C1 cells expressing *siCCNE1* or siCtrl. Performed in triplicate. *P <0.001, Student’s *t* test. (**R**) Adavosertib IC_50_ dose in NCC_CDS1_X1_C1 cells expressing si*CCNE1*, si*CCNE2*, si*CCNE1*, and si*CCNE2* compared with siCtrl; performed in triplicate. *P <0.001, 1-way ANOVA.

Prior studies have identified cell cycle regulators as direct transcriptional targets of the CIC-DUX4 fusion oncoprotein (4, 18, 19). One key CIC-DUX4 transcriptional target is *CCNE1*, which regulates the G_1_/S cell cycle transition (4, 20). CIC-DUX4–dependent *CCNE1* transcriptional upregulation compromises the G_1_/S checkpoint and confers molecular and therapeutic dependence on the CCNE1/CDK2 complex (4). In order to identify additional actionable therapeutic targets in CIC-DUX4 sarcomas, we integrated CIC-DUX4–dependent gene expression changes with functional kinase activity screens in cells derived from patients with CIC-DUX4 sarcoma to identify candidate kinases that enable CIC-DUX4 survival (Figure 1A). Specifically, we performed comparative transcriptional analysis using a validated data set (GSE60740) comprising cells derived from patients with CIC-DUX4 sarcoma (IB120) with or without genetic silencing of CIC-DUX4 (19). Through a previously described approach in which IB120 cells with and without CIC-DUX4 expression were compared (4), we identified 165 putative CIC-DUX4–responsive genes, 43 of which contained the highly conserved CIC-binding motif (T[G/C]AATG[A/G]A) within –2 kb and +150 bp of the transcription start site (21) (see Methods for details). This analysis enabled us to identify high-confidence CIC-DUX4–dependent gene expression changes in cells endogenously expressing CIC-DUX4. In concordance with prior findings, we noted significant enrichment in genes that regulate the cell cycle, including G_1_/S transition, mitosis, and cell cycle checkpoints (4). Genes that regulate DNA replication and chromosome segregation were also enriched in CIC-DUX4–replete cells compared with CIC-DUX4–KD cells (Figure 1B). In order to confirm these findings, we performed unbiased gene set enrichment analysis (GSEA) using 1426 up- and down-regulated genes identified upon CIC-DUX4 KD in IB120 (endogenous CIC-DUX4) cells (Supplemental Table 1; supplemental material available online with this article; https://doi.org/10.1172/jci.insight.152293DS1). This analysis consistently demonstrated enrichment of gene sets associated with G_1_/S, mitosis, cell cycle checkpoints, and DNA replication (Figure 1C). These expression data indicated that cell cycle and DNA replication programs are key molecular targets of the CIC-DUX4 fusion oncoprotein. Next, we queried a publicly available data set that had been subjected to multiplex kinase activity (PamGene) profiling to identify active kinases in patient-derived CIC-DUX4 tumors, xenografts, and cell lines (22). Since our transcriptional analysis converged on genes involved in DNA replication, G_1_/S transition, and mitosis, we focused on kinases that regulate the cell cycle in CIC-DUX4 sarcoma. Through a manual systematic analysis, we identified 20 unique phosphosites mapping to 14 independent kinases previously implicated in cell cycle regulation (Figure 1D) (22). Multiple CDK1 (4) and CDK2 (3) phosphosites were identified in our analysis, suggesting that these kinases have broad roles in CIC-DUX4 sarcoma cells. We noted that phosphosites mapping to the WEE1 kinase were highly repetitive and specific for the inhibitory phosphorylation site (Y15) on both CDK1 and CDK2, suggesting that CIC-DUX4 cells may depend on WEE1 activity to potentially delay G_2_/M progression, and limit premature mitotic entry and mitotic catastrophe (Figure 1, E and F) (13, 15, 17). Consistent with this, we previously noted that ectopic expression of CIC-DUX4 leads to an increased G_2_/M fraction in NIH 3T3 cells (4). Since CIC-DUX4 transcriptionally upregulates *CCNE1*, which can induce a high-replicative-stress state in cancer (5, 6, 9–11, 23), we hypothesized that WEE1 activity in CIC-DUX4 sarcomas may be an adaptive survival mechanism. To test whether WEE1 is necessary for CIC-DUX4 survival, we treated 2 independent patient-derived CIC-DUX4 cell lines (NCC_CDS1_X1_C1 and NCC_CDS2_C1) (22, 24) with the WEE1 inhibitor adavosertib (AZD1775) (25–28). Adavosertib treatment significantly decreased the viability of both NCC_CDS1_X1_C1 and NCC_CDS2_C1 cells as measured by CellTiter-Glo (CTG) and crystal violet assays (Figure 2, A–C). The decreased viability observed upon adavosertib treatment was associated with a decrease in CDK1 phosphorylation at Y15 and an increase in apoptosis as measured by poly(ADP-ribose) polymerase (PARP) cleavage in CIC-DUX4–expressing NCC_CDS_X1_C1 and NCC_CDS2_C1 cells. Moreover, we observed an increase in phosphorylation of serine 139 on the histone variant γH2AX, a sensitive marker of DNA damage, in adavosertib-treated compared with control cells (Figure 2, D and E) (29). Coupled with the enhanced caspase-3/7 activity observed upon adavosertib treatment, these findings indicate that pharmacologic inhibition of WEE1 induces apoptosis in CIC-DUX4 sarcoma cells, potentially through increased DNA damage and premature mitotic death (Figure 2, F and G). To further mitigate possible off-target effects of adavosertib, we performed genetic silencing of *WEE1* using 2 validated siRNAs (siWEE1-06 and siWEE1-08) that target independent regions of *WEE1* (30). Consistent with our pharmacologic findings, genetic inhibition of WEE1 decreased CDK1 Y15 phosphorylation and resulted in enhanced γH2AX expression and PARP cleavage compared with control (Figure 2, H and I). Moreover, genetic silencing of WEE1 decreased NCC_CDS1_X1_C1 and NCC_CDS2_C1 cell viability compared with control as measured by CTG assay (Figure 2, J and K) and crystal violet (Figure 2, L and M) assays. In contrast, we did not observe a decrease in the viability of A673 (Ewing sarcoma [ES], EWSR1-FLI1^+^) or RH41 (alveolar rhabdomyosarcoma, PAX3-FOXO1^+^) cells upon WEE1 KD compared with control (Supplemental Figure 1, A–D). In order to further demonstrate that the adavosertib-mediated apoptotic effect was dependent on CDK1 and/or CDK2, we silenced CDK1 and/or CDK2 in CIC-DUX4–expressing cells. We noted an increase in IC_50_ with combinatorial CDK1 and CDK2 silencing relative to control or either CDK1 or CDK2 KD alone (Figure 2, N and O, and Supplemental Figure 2, A–F). In order to further link WEE1-mediated suppression of CDK1 activity to survival, we used the well-characterized CDK1 variant CDK1^AF^ (31). The WEE1 inhibitory phosphosite in CDK1 (Y15) is replaced in CDK1^AF^, generating a constitutively active isoform that is not responsive to WEE1 activity. Expression of this CDK1^AF^ variant in NCC_CDS1_X1_C1 or NCC_CDS2_C1 cells decreased viability compared with control (Figure 2P). These findings indicate that WEE1 may promote CIC-DUX4 survival through inhibitory phosphorylation of CDK1. Through these studies, we reveal a pro-survival role for WEE1 and highlight a therapeutic vulnerability in CIC-DUX4 sarcomas.

Multiple preclinical studies have recently shown that in vitro efficacy of adavosertib does not directly translate into clinical responses to WEE1 inhibition in unselected patient populations. Importantly, increased *CCNE1* expression has been consistently correlated with clinical response to WEE1 inhibitors (32–37). Thus, *CCNE1* mRNA expression is a patient selection biomarker for WEE1 inhibitor response in patients with cancer at the clinical level. Therefore, in order to understand whether WEE1 inhibitor response is mechanistically linked to CCNE1 expression in CIC-DUX4–positive cell lines, we first knocked down *CCNE1* in NCC_CDS1_X1_C1 and NCC_CDS2_C1 cells and treated them with adavosertib. We observed decreased sensitivity to adavosertib in NCC_CDS2_C1 cells expressing si*CCNE1* compared with those expressing si*Control* (Figure 2Q). Moreover, since we previously noted a compensatory increase in *CCNE2* (>100-fold) expression in NCC_CDS1_X1_C1 cells upon *CCNE1* KD (4), we performed dual *CCNE1* and *CCNE2* KD in NCC_CDS1_X1_C1 cells; we consistently noted a decrease in adavosertib sensitivity, as demonstrated by IC_50_ dose compared with control (Figure 2R). We did not observe a similar increase in adavosertib IC_50_ dose or *CCNE2* mRNA expression in A673 (ES, EWSR1-FLI1^+^) and RH41 (alveolar rhabdomyosarcoma, PAX3-FOXO1^+^) cells upon *CCNE1* KD (Supplemental Figure 3, A–F). These findings indicated that the effect of the WEE1 inhibitor that we observed in NCC_CDS1_X1_C1 and NCC_CDS2_C1 cells was partially dependent on CCNE1/CCNE2 expression. Since CCNE1 expression is a clinically validated biomarker of WEE1 inhibitor response, we anticipate that individuals with CIC-DUX4 sarcomas, but not other small round blue cell tumors including ES or alveolar rhabdomyosarcoma, will benefit from WEE1 inhibitor treatment.

**Figure 3:**
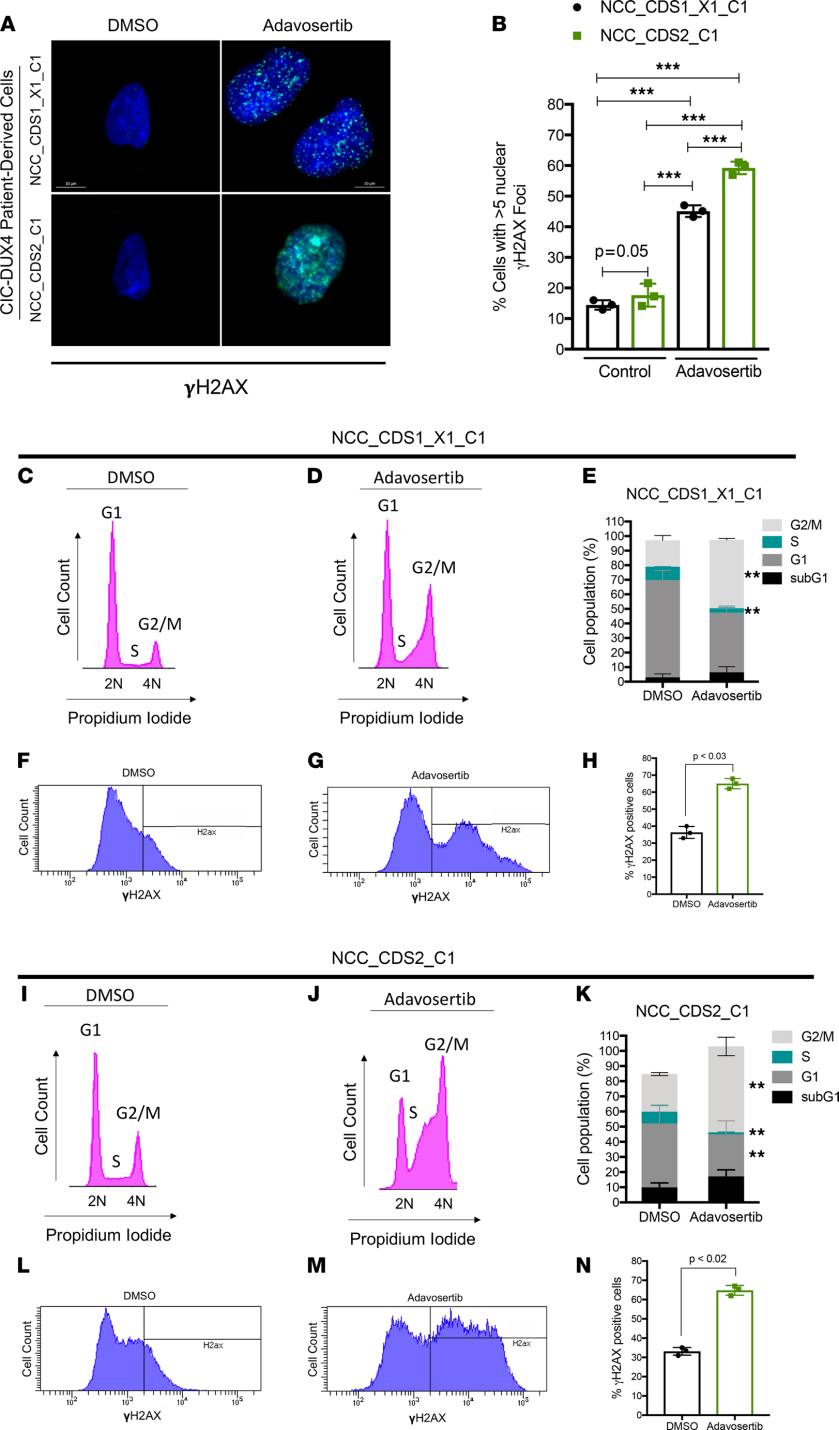
WEE1 inhibition increases DNA damage and mitotic entry in CIC-DUX4 sarcoma cells. (**A**) γH2AX IF staining of NCC_CDS1_X1_C1 and NCC_CDS2_C1 cells treated with adavosertib (0.5 μM) or DMSO (48 hours). Representative figure; performed in triplicate. Scale bars: 10 μm. (**B**) Quantitative analysis of γH2AX IF in **A** demonstrating the percentage of NCC_CDS1_X1_C1 or NCC_CDS2_C1 cells with more than 5 nuclear γH2AX foci following treatment with adavosertib or control. Mean percentage over 10 HPFs. ***P < 0.001, 1-way ANOVA. Representative cell cycle histograms (PI staining) of NCC_CDS1_X1_C1 (**C** and **D**) and NCC_CDS2_C1 (**I** and **J**) cells treated with adavosertib or DMSO (48 hours). Percentage of adavosertib- (0.5 μM) and DMSO-treated (48 hours) NCC_CDS1_X1_C1 (**E**) and NCC_CDS2_C1 (**K**) cells in sub-G_1_, G_1_, S, and G_2_/M phases as identified in **C**, **D**, **I**, and **J**. **P < 0.05, 1-way ANOVA. Performed in triplicate. Error bars represent SEM. Values for each fraction of cells in sub-G_1_, G_1_, S, and G_2_/M are provided in Supplemental Figure 4, A and B. γH2AX expression in NCC_CDS1_X1_C1 (**F** and **G**) and NCC_CDS2_C1 (**L** and **M**) cells treated with adavosertib or DMSO. Percentage of γH2AX-positive cells among NCC_CDS1_X1_C1 (**H**) and NCC_CDS2_C1 (**N**) cells analyzed in **F**, **G**, **L**, and **M**. Student’s *t* test. Error bars represent SEM.

In certain cancers, increased CCNE1 expression (amplification or transcriptional upregulation) deregulates the cell cycle at the G_1_/S and G_2_/M checkpoints by accelerating S-phase entry and stimulating premature mitosis (5, 7, 9–11, 23). Moreover, a hyperactive CCNE1/CDK2 complex can increase DNA origin firing–inducing re-replication, leading to high DNA replication stress — a state that requires adaptive mechanisms at the cellular level to enable survival (38, 39). One response to this oncogene-induced replicative stress involves an increased dependence WEE1 (15, 16). Since CIC-DUX4 directly binds to the regulatory region of *CCNE1* to hyperactivate the CCNE1/CDK2 complex (4), we hypothesized that CIC-DUX4–mediated *CCNE1* upregulation induces DNA replication stress, stimulating DNA repair responses and thus sensitizing to WEE1 inhibition. To mechanistically dissect the role of WEE1 in limiting extensive DNA damage and premature mitotic entry, we first quantified γH2AX nuclear foci in NCC_CDS1_X1_C1 and NCC_CDS2_C1 cells through immunofluorescence (IF) staining following adavosertib treatment, as previously described (11). We noted an increased fraction of cells with a high degree of staining for nuclear γH2AX foci (>5 foci per cell) in adavosertib-treated compared with control cells (Figure 3, A and B). These findings indicated that WEE1 activity can limit DNA damage in CIC-DUX4 sarcomas. Since WEE1 inhibition with adavosertib induces premature mitotic entry and DNA damage (40, 41) in other cancer types, we next determined the effect of adavosertib on cell cycle progression in our patient-derived CIC-DUX4 sarcoma cells. Specifically, we analyzed the cell cycle distribution and γH2AX expression in NCC_CDS1_X1_C1 and NCC_CDS2_C1 cells in response to adavosertib treatment. Compared with control, adavosertib (0.5 μM at 48 hours) increased the G_2_/M fraction in our CIC-DUX4 cell lines (Figure 3, C–E and I–K, and Supplemental Figure 4, A and B), thus indicating premature mitotic entry and/or mitotic arrest that was associated with extensive DNA damage, as measured by increased γH2AX expression and an increase in the fraction of polyploid (>4N) cells (Figure 3, F–H and L–N, and Supplemental Figure 4, C–F). Consistent with our prior studies, we also observed an increase in the sub-G_1_ fraction of adavosertib-treated compared with control cells (Supplemental Figure 4, C–F). Collectively, these findings demonstrate that WEE1 inhibition with adavosertib leads to premature mitosis, DNA damage, and apoptotic cell death.

**Figure 4:**
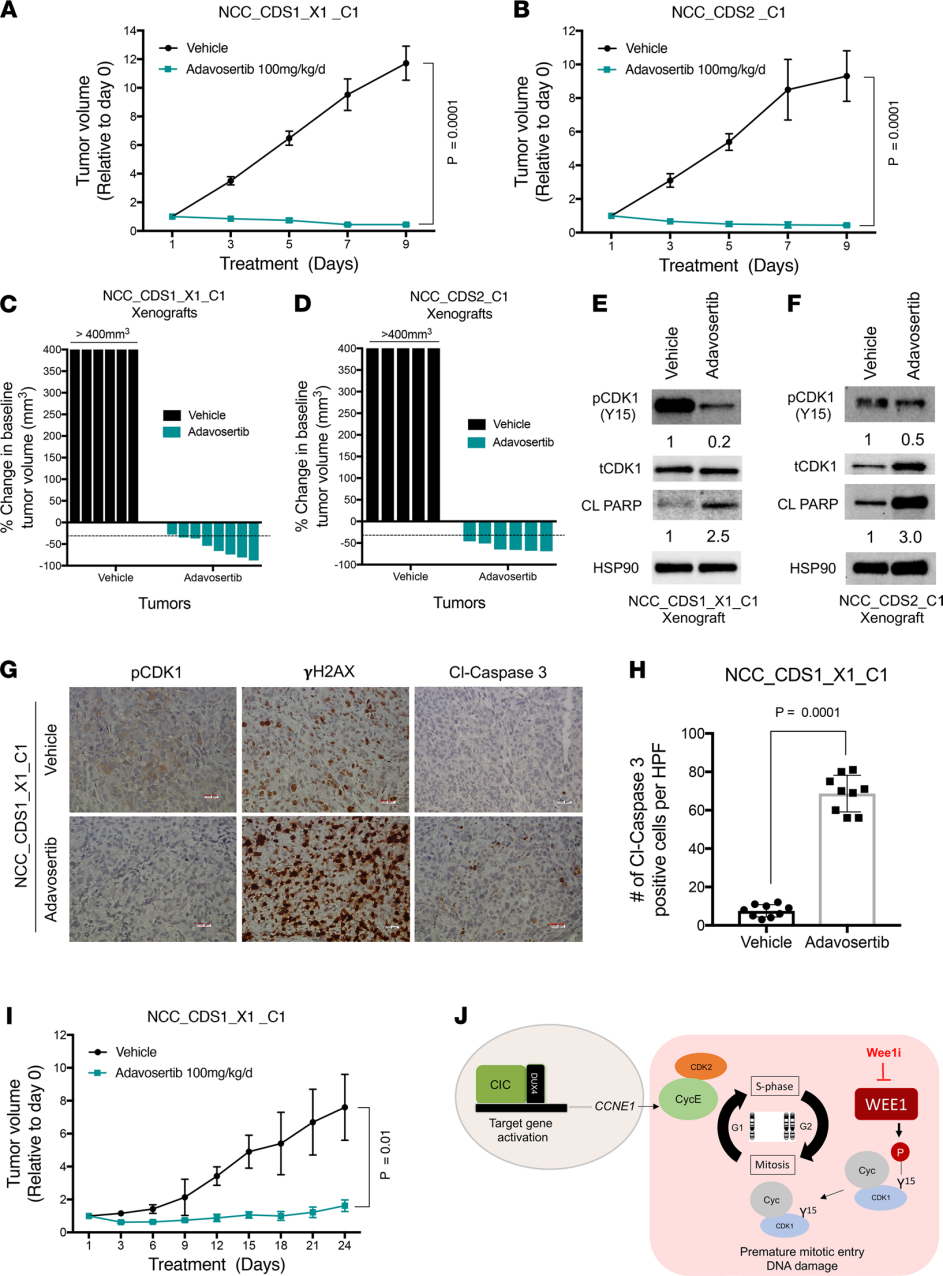
WEE1 is a therapeutic target in CIC-DUX4 sarcomas. Relative tumor volume of NCC_CDS1_X1_C1 (**A**) (*n* = 8 for adavosertib and *n* = 6 for vehicle control groups) and NCC_CDS2_C1 (**B**) (*n* = 6 for adavosertib and *n* = 5 for vehicle control groups) treated with adavosertib or vehicle control. Student’s *t* test. Error bars represent SEM. (**C** and **D**) Percent change from baseline in tumor volume for NCC_CDS1_X1_C1 (*n* = 8 for adavosertib and *n* = 6 for vehicle control group) and NCC_CDS2_C1 (*n* = 6 for adavosertib and *n* = 5 for vehicle control) tumor–bearing mice in the adavosertib and vehicle cohorts. Percent change from baseline tumor volume for each mouse is shown in Supplemental Figure 5, C and D. (**E** and **F**) Immunoblot of NCC_CDS1_X1_C1 and NCC_CDS2_C1 tumor explants treated with adavosertib or vehicle control. (**G**) Representative IHC images of pCDK1, γH2AX, and cleaved caspase-3 in NCC_CDS1_X1_C1 tumor xenografts derived from mice treated with adavosertib or vehicle control. Scale bars: 100 μm. (**H**) Number of cleaved caspase-3–positive cells per HPF; 9 HPFs analyzed. Student’s *t* test. (**I**) Tumor volume of NCC_CDS1_X1_C1 xenograft–bearing mice treated for 24 days with adavosertib (*n* = 6) compared with vehicle control (*n* = 6). (**J**) Model: CIC-DUX4–regulated *CCNE1* transcriptional upregulation leads to survival dependence on the G_2_/M checkpoint kinase WEE1. Wee1i, WEE1 inhibition.

CIC-DUX4 sarcomas are universally associated with poor clinical outcomes due to rapid metastatic progression and insensitivity to conventional chemotherapy agents (42). Thus, we next tested the translational impact of targeting the WEE1 kinase in preclinical models of CIC-DUX4 sarcoma. Specifically, we generated NCC_CDS1_X1_C1 and NCC_CDS2_C1 tumor xenografts in immunodeficient mice and treated them with adavosertib (100 mg/kg/d) and or vehicle control. In NCC_CDS1_X1_C1– and NCC_CDS2_C1–bearing mice, we noted tumor regression in the adavosertib compared with the vehicle-treated group (Figure 4, A and B, and Supplemental Figure 5, A–D). The overall objective response rates (≥30% reduction in tumor volume) to adavosertib in the NCC_CDS1_X1_C1 and NCC_CDS2_C1 cohorts were 87.5% (7 of 8) and 100% (6 of 6), respectively, without noted toxicity (Figure 4, C and D, and Supplemental Figure 6, A and B). Consistent with our in vitro data, NCC_CDS1_X1_C1 and NCC_CDS2_C1 tumor explants from adavosertib-treated mice demonstrated a decrease in CDK1 phosphorylation (Y15) and increased PARP cleavage compared with vehicle control (Figure 4, E and F). Immunohistochemical (IHC) analysis of NCC_CDS1_X1_C1 tumor explants further validated that WEE1 inhibition increased DNA damage and enhanced apoptosis, as demonstrated by increased γH2AX and cleaved caspase-3 expression in mice treated with adavosertib compared with vehicle control (Figure 4, G and H). To further assess the durability of the adavosertib response, we treated NCC_CDS1_X1_C1 tumor–bearing mice with adavosertib (100 mg/kg/d) for 24 days. Following initial tumor regressions, we observed prolonged tumor growth suppression (Figure 4I). These in vivo studies further validated WEE1 as a therapeutic vulnerability in CIC-DUX4 sarcomas that can be readily targeted through clinically advanced WEE1 inhibitors, including adavosertib (28, 35). Collectively, these data demonstrate a mechanism-based therapeutic strategy to precisely and effectively target CIC-DUX4 sarcomas in patients.

In this study, we employed an integrative transcriptional and functional approach to identify key clinically actionable vulnerabilities in CIC-DUX4 sarcoma. Through this analysis, we identified a WEE1-mediated adaptive response that enables CIC-DUX4 sarcoma survival by limiting massive DNA damage and mitotic catastrophe. These findings are in line with and expand our prior studies that reveal a dependence on CIC-DUX4–driven *CCNE1* expression in CIC-DUX4 sarcomas (4). Thus, we demonstrate that CIC-DUX4 sarcomas transcriptionally upregulate *CCNE1*, compromising the G_1_/S checkpoint and conferring dependence on WEE1 to limit DNA damage–associated cell death (Figure 4J). Importantly, these studies reveal a precision medicine–based therapy for CIC-DUX4 sarcomas, which remain an aggressive and lethal subset of human cancer.

## Discussion

Through our studies we provide the initial translational framework for use of WEE1 inhibition as a therapeutic strategy in CIC-DUX4 sarcomas. Future studies should focus on rational combinatorial strategies to enhance DNA damage and potentially augment WEE1 inhibitor responses. Adavosertib is currently being evaluated in multiple tumor histologies and is proven safe as a monotherapy or in combination with other conventional targeted and chemotherapeutic agents as well as radiotherapy (11, 27, 28, 35). Importantly, our preclinical findings also illustrate how pharmacologic manipulation of key cell cycle regulators (CDK1 and/or CDK2) can potentially limit the efficacy of WEE1 inhibitors in cancer patients. With this caveat, adavosertib therapy is potentially a safe and efficacious therapy (28) that can be rapidly employed in the clinic to effectively target CIC-DUX4 sarcomas. Similarly, ZN-c3 (WEE1 inhibitor; Zentalis) is entering phase II studies as monotherapy and/or combination therapy in solid tumors, including sarcomas. One barrier in exploring the clinical response to WEE1 inhibitors in CIC-DUX4 patients is the relative rarity of CIC-DUX4 sarcomas (43). To overcome this obstacle, the sarcoma community must first differentiate CIC-DUX4 sarcomas as a unique entity and not routinely integrate them (clinically or pathologically) into more common small round cell sarcoma subtypes such as ES (4, 18, 20, 42, 44). This misrepresentation leads to a misconception that CIC-DUX4 sarcomas should be managed and treated with a strategy similar to that for ES (42, 45, 46). In most cases, ES-directed chemotherapy is not effective in CIC-DUX4 sarcomas (42). Thus, in order to advance the field, we must develop a more rational approach to treat this ultra-rare yet lethal subtype. Additionally, extensive collaboration is warranted to more rapidly identify and direct patients with CIC-DUX4 sarcoma to clinical trials that may be effective, such as WEE1 inhibitors (monotherapy or combination) or CDK2-directed therapies, as previously described (4). Finally, perhaps a reevaluation of clinical trial end points and a centralized clinical center for rare cancers would enhance clinical collaboration and accelerate therapeutic advancements (43).

## Methods

### Tumor xenografts.

Six- to eight-week-old female nude (NU/J) mice were purchased from the Jackson Laboratory. Specific pathogen–free conditions and facilities were approved by the American Association for Accreditation of Laboratory Animal Care. For subcutaneous xenotransplantation, 1.5 × 10^6^ NCC_CDS1_X1_C1 and NCC_CDS2_C1 cells were resuspended in 50% PBS/50% Matrigel matrix and injected into the flanks of immunodeficient (NU/J) mice. Tumor-bearing mice were treated with either adavosertib or vehicle control for the designated time points.

### Cell lines and culture reagents.

NCC_CDS1_X1_C1 and NCC_CDS2_C1 were generated as patient-derived cell lines by a member of our research team (22, 24). The presence of the CIC-DUX4 fusion was confirmed in NCC_CDS1_X1_C1 and NCC_CDS2_C1 cells through RNA-Seq analysis using the “grep” command as previously described (47). NCC_CDS1_X1_C1 cells were maintained at 37°C in a humidified atmosphere at 5% CO_2_ and grown in RPMI 1640 medium supplemented with 10% FBS, 100 IU/mL penicillin, and 100 μg/mL streptomycin. NCC_CDS2_C1 cells were cultured in RPMI medium supplemented with 10% FBS, 100 IU/mL penicillin, and 100 μg/mL streptomycin. Adavosertib (AZD1775, MK1775) was purchased from MedChemExpress (HY-10993).

### Gene KD and overexpression assays.

ON-TARGET scramble, WEE1 (WEE11-06-J-005050-06 and WEE1-08-J005050-08), CDK2 (L-003236-00-0005), CDK1 (L-003224-00-0005), CCNE1 (L-003213-00-0005), and CCNE2 (L-003214-00-0005) siRNAs were obtained from GE Dharmacon, and transfections were performed with DharmaFECT transfection reagent. CDK1 (catalog 61840) and CDK1^AF^ (catalog 39872) plasmids were purchased from Addgene, and transfections were performed with FuGENE 6 transfection reagent.

### Western blot analysis.

All immunoblots represent at least 2 independent experiments. Adherent cells were washed and lysed with RIPA buffer supplemented with proteinase and phosphatase inhibitors. Proteins were separated by SDS-PAGE, transferred to nitrocellulose membranes, and blotted with Cell Signaling Technology (CST) antibodies recognizing HSP90 (CST 4874), total CDK1 (CST 9116), phosphorylated (Y15) CDK1 (CST 9111), WEE1 (CST 13084), PARP (CST 9542), total H2AX (CST 7631), and phosphorylated (S139) γH2AX (CST 9718).

### Real-time quantitative PCR.

Isolation and purification of RNA were performed using an RNeasy Mini Kit (QIAGEN). Total RNA (500 ng) was used in a reverse transcriptase reaction with the SuperScript III First-Strand Synthesis System (Invitrogen). Quantitative PCR included 3 replicates per cDNA sample. Human CDK1, CDK2, CCNE1, CCNE2, and GAPDH were amplified with Taqman gene expression assays (Applied Biosystems). Expression data were acquired using an ABI Prism 7900HT Sequence Detection System (Applied Biosystems). Expression of each target was calculated using the 2^–ΔΔCt^ method, and mRNA levels are expressed relative to GAPDH.

### Xenograft tumor preparation for Western blot analysis.

Subcutaneous xenografts were explanted at the study end point and immediately immersed in liquid nitrogen and stored at –80°C. Tumors were disrupted with a mortar and pestle, followed by sonication in RIPA buffer supplemented with proteinase and phosphatase inhibitors. Proteins were separated as above. Antibodies to PARP, phosphorylated (Y15) CDK1, HSP90, and total CDK1 were purchased from CST.

### Xenograft tumor preparation for IHC staining.

Mice bearing NCC_CDS1_X1_C1 tumor xenografts were treated with 100 mg/kg adavosertib or vehicle control for 4 consecutive days. On day 4, tumors were explanted, fixed in 10% neutral buffered formalin for 72 hours, and embedded in paraffin, and sections of 5–10 μm were prepared. Sections were subsequently deparaffinized and incubated with antibodies directed against pCDK1 (CST 9111), γH2AX (CST 9718), and cleaved caspase-3 (CST 9661) overnight. Quantification of cleaved caspase-3 IHC staining was performed through analysis of at least 8 high-power fields (HPFs) per condition. Images were captured on a Zeiss Axioplan II microscope.

### Cell viability assays.

Cells were seeded overnight at a density of 3000 cells per well in 96-well plates and treated with relevant agents for 72 hours. Cell viability was determined using the CTG (Promega) assay according to the manufacturer’s protocol. Each assay consisted of at least 3 replicate wells. For crystal violet assays, 100,000 cells were seeded per well in a 12-well plate (250,000 cells in a 6-well plate) and allowed to grow for 5 consecutive days. Cells were then fixed in 4% paraformaldehyde, followed by 0.05% crystal violet staining. Quantification was performed using ImageJ (NIH) software.

### Apoptosis assays.

Cells were seeded overnight at a density of 40,000 cells per well in 96-well plates and treated with relevant agents for 24 hours. Caspase-3/7 activity was measured on a Molecular Devices microplate reader using Caspase-Glo reagent per the manufacturer’s protocol (Promega) and normalized to cell number.

### IF.

IF was performed on glass coverslips. Cells were fixed with 4% paraformaldehyde, quenched with 1× PBS and 10 mM glycine, and permeabilized with 0.1% Triton X-100, then incubated with conjugated (Alexa Fluor 488) phospho–histone H2A.X (S139) antibody (CST). ProLong Gold Antifade Mountant (Thermo Fisher Scientific) with DAPI was applied directly to fluorescently labeled cells on microscope slides. Fluorescence images were collected on a Zeiss Axioplan II fluorescence microscope.

### Cell cycle analysis.

To determine the effect of adavosertib (0.5 μm for 48 hours) on the cell cycle of NCC_CDS1_X1_C1 and NCC_CDS2_C1 cells, we first trypsinized them, washed with PBS plus 0.1% BSA, and fixed in ice-cold ethanol overnight. We subsequently treated them with RNase (CST) and stained with propidium iodide (PI) solution (Thermo Fisher Scientific) or with conjugated (Alexa Fluor 488) phospho–histone H2A.X (S139) antibody at room temperature for 30 minutes. Cells were analyzed on a BD LSR II flow cytometer.

### Pathway analysis and GSEA.

As previously described, 165 downregulated genes were identified in IB120 cells expressing siCtrl or siCIC-DUX4. 43 of 165 putative gene targets contained the CIC DNA-binding motif (T[G/C]AATG[A/G]A) within –2 kb and +150 bp of the transcription start site (4). These 43 high-confidence genes were analyzed using Reactome Pathway software (https://reactome.org/) to identify CIC-DUX4–regulated pathways. GSEA was performed using the top 1426 up-and downregulated genes in IB120 cells expressing siCIC-DUX4 versus control.

### Kinase array analysis.

As previously described, multiplex kinase activity profiling was performed in cells derived from patients with CIC-DUX4 sarcoma using PamGene technology and measured by PamStation according to the manufacturer’s protocol (22). Manual identification of cell cycle kinases through the phosphorylation status of substrate peptides was performed using the data set published by Oyama et al. (22).

### Statistics.

Experimental data are presented as mean ± SEM. *P* values derived for all in vitro and in vivo experiments were calculated using 2-tailed Student’s *t* test or 1-way ANOVA. A *P* value less than 0.05 was considered significant.

### Study approval.

Tumor xenotransplantion and animal surgical procedures were reviewed and approved by the UCSF IACUC, protocol AN178670-03B

## Author contributions

RKMP and NJT designed and performed the experiments and analyzed the data. NQB analyzed the data and provided critical revisions on the manuscript. TK performed experiments and provided cell lines. RAO directed the project, analyzed experiments, and wrote the manuscript.

## Supplementary Material

Supplemental data

Supplemental table 1

## Version 1 03/22/2022

Electronic publication
