# Supplementary material for: WEE1 kinase is a therapeutic vulnerability in CIC-DUX4 undifferentiated sarcoma
Source: JCI Insight. 2022 Mar 22;7(6):e152293. doi: 10.1172/jci.insight.152293 (PMC8986087; doi:10.1172/jci.insight.152293)
Supplement: Supplemental data [file jciinsight-7-152293-s005.pdf]

Supplemental material

WEE1 kinase is a therapeutic vulnerability in CIC-DUX4 undifferentiated sarcoma.

Authors: Rovingaile Kriska Ponce, Nicholas J. Thomas, Nam Q. Bui, Tadashi Kondo, and Ross A. Okimoto

The Supplemental material contains 6 figures and one table.

Supplementary Figure 1

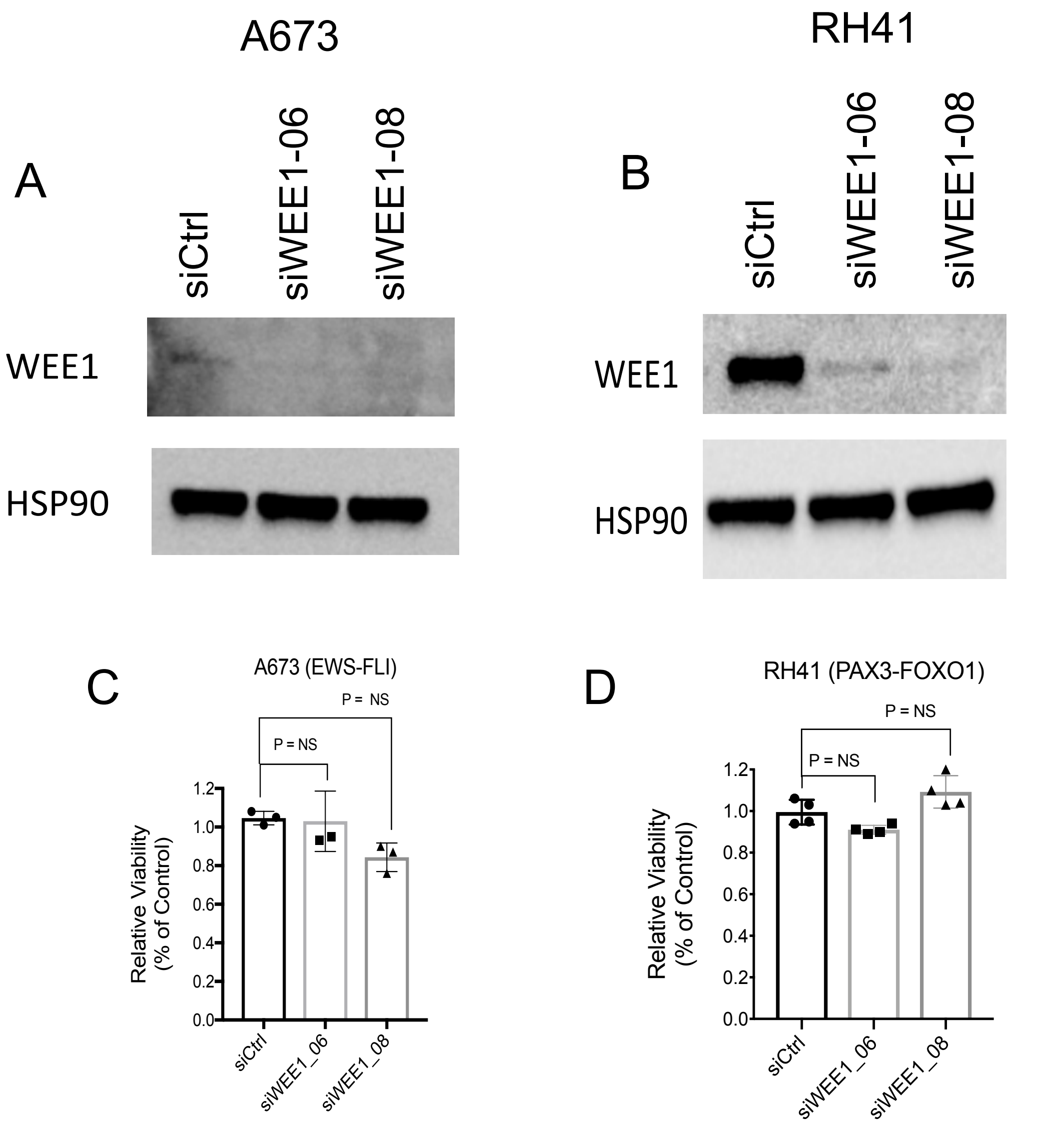

Supplementary Figure 1. Genetic WEE1 inhibition in Ewing sarcoma and alveolar rhabdomyosarcoma cells. (A) Immunoblot of WEE1 and HSP90 in A673 (A) and RH41 (B) cells expressing *siWEE1*-06 or *siWEE1*-08 compared to siCtrl. (B) Relative viability (CTG) of A673 (C) and RH41 (D) cells expressing *siWEE1*-06 or *siWEE1*-08 compared to *siCtrl.* Performed in quadruplicate. P-value calculated by 1-way ANOVA.

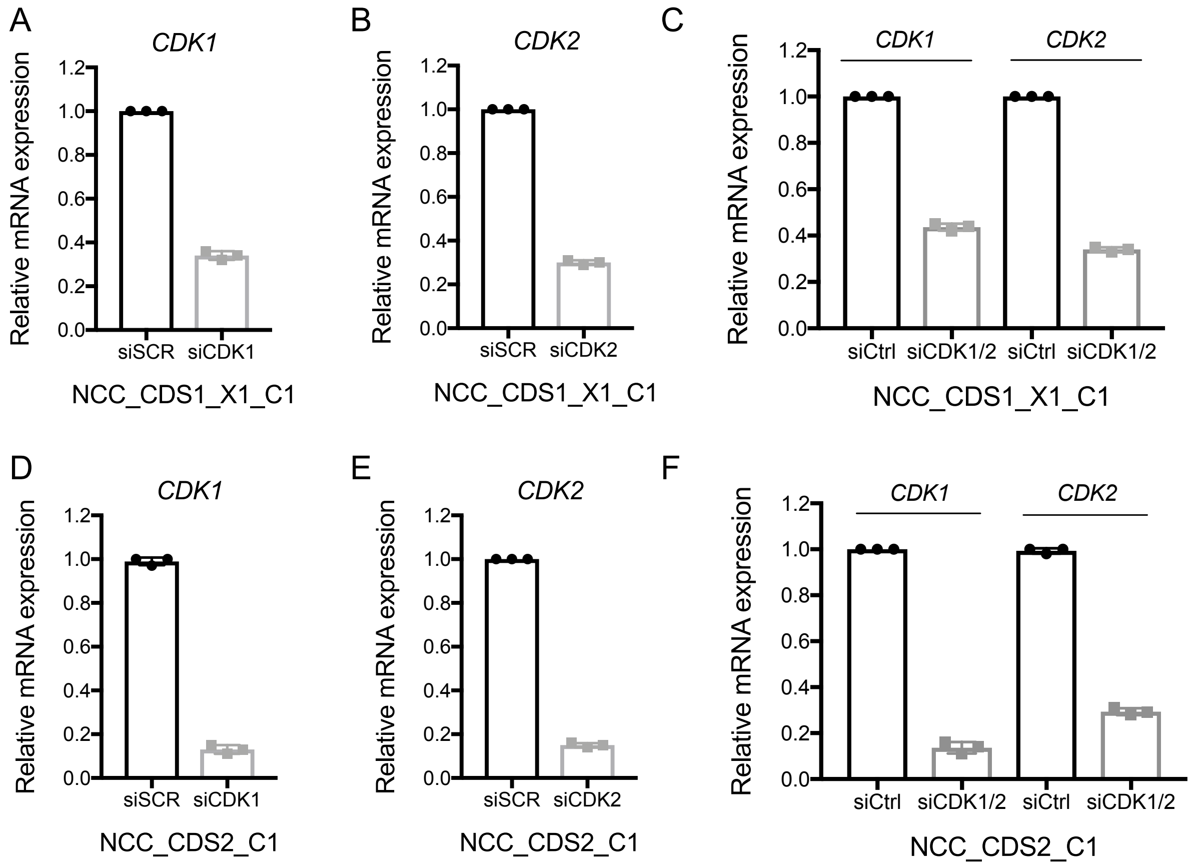

Supplementary Figure 2. CDK1 and CDK2 genetic silencing in CIC-DUX4 cells.

Relative mRNA expression of CDK1 (A), CDK2 (B), and CDK1 and CDK2 (C) in NCC_CDS1_X1_C1 cells following siRNA-mediated knockdown. Relative mRNA expression of CDK1 (D), CDK2 (E), and CDK1 and CDK2 (F) in NCC_CDS2_C1 cells following siRNA-mediated knockdown.

Supplementary Figure 3

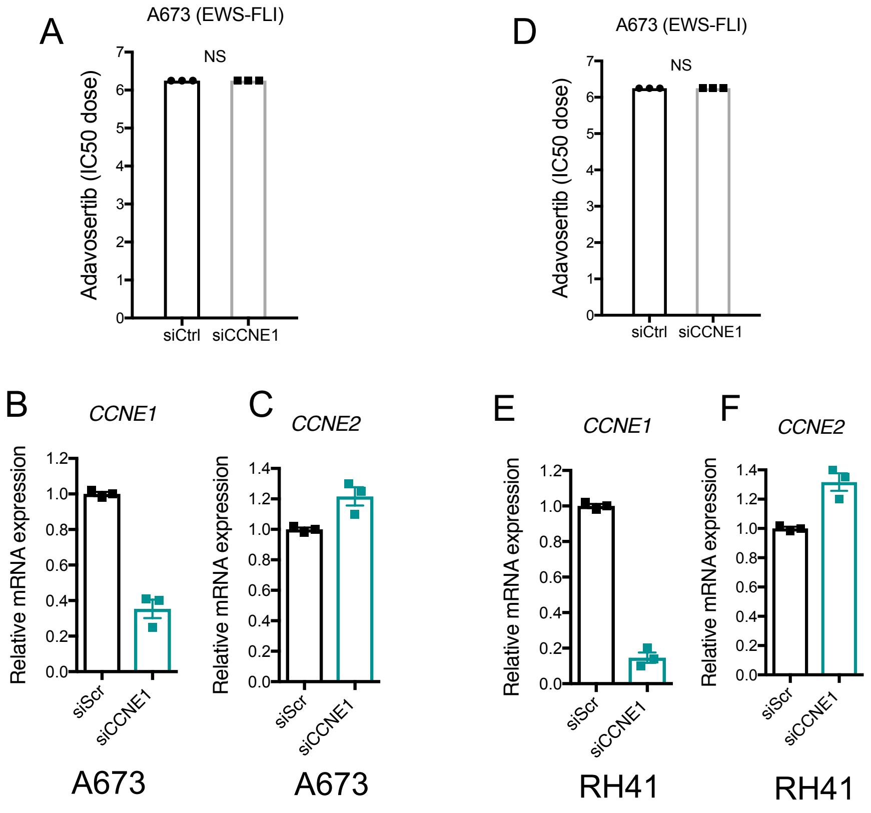

Supplementary Figure 3. Sensitivity to adavosertib treatment is not dependent on CCNE1 expression in Ewing sarcoma and alveolar rhabdomyosarcoma cells. Adavosertib IC50 dose of A673 (A) and RH41 (D) cells expressing *siCCNE1* or *siScr*. Performed in quadruplicate. P-value calculated by student's t-test. Relative *CCNE1* (B) and *CCNE2* (C) mRNA expression in A673 cells expressing either *siCCNE1* or *siCtrl*. Performed in triplicate. P-value calculated by student's t-test. Relative *CCNE1* (E) and *CCNE2* (F) mRNA expression in RH41 cells expressing either *siCCNE1* or *siCtrl.* Performed in triplicate. P-value calculated by student's t-test.

A

NCC_CDS1_X1_C1												
Cell Cycle	%SubG1	%SubG1	%SubG1	%G1	%G1	%G1	%S	%S	%S	%G2/M	%G2M	%G2/M
DMSO	1.47	5.72	1.8	59.2	71	70	9.2	9.2	9.3	21.5	15	18
Adavosertib	2.32	10	7	36.6	46	40	3.83	2.5	3	48	46	47

B

NCC_CDS2_C1												
Cell Cycle	%SubG1	%SubG1	%SubG1	%G1	%G1	%G1	%S	%S	%S	%G2/M	%G2M	%G2/M
DMSO	4.8	15	10	30.6	54	42	3.37	12	8	25.88	24	24.5
Adavosertib	9.8	24.7	17	36.6	18.7	28	1.54	0.4	1	51.54	63.6	56

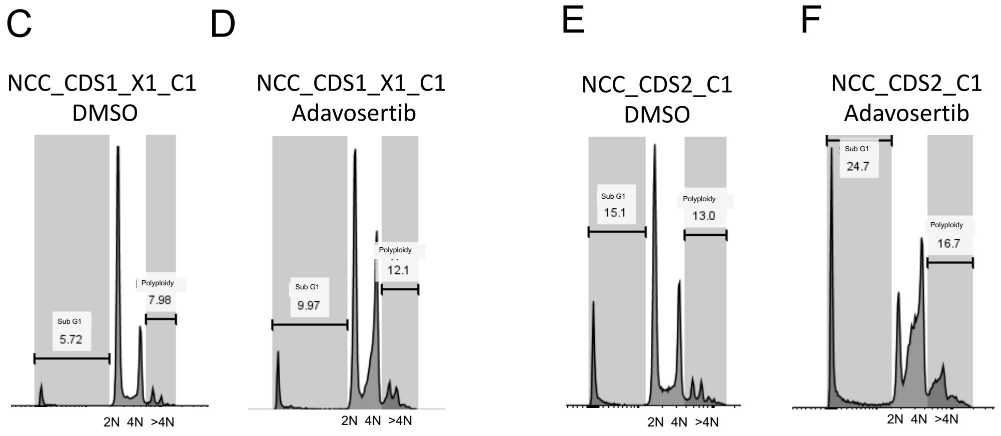

Supplementary Figure 4. Pharmacologic WEE1 inhibition increases the G2/M, polyploidy (>4N), and sub G1 fractions in CIC-DUX4 sarcoma cells.

(A) Individual data points from figure 3E demonstrating the fraction of cells in Sub-G1, G1, S, or G2/M phases of the cell cycle. (B) Individual data points from figure 3K demonstrating the fraction of cells in Sub-G1, G1, S, or G2/M phases of the cell cycle.

Representative figures of ungated cell cycle analysis profiles of NCC_CDS1_X1_C1 cells treated with adavosertib (C) or DMSO (D) control. Performed in triplicate. Representative figures of ungated cell cycle analysis profiles of NCC_CDS2_C1 cells treated with adavosertib (E) or DMSO (F) control. Performed in triplicate.

Supplementary Figure 5

A

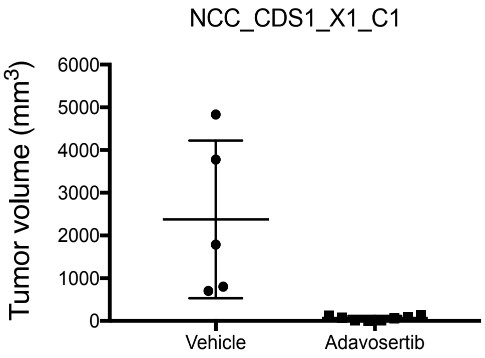

B

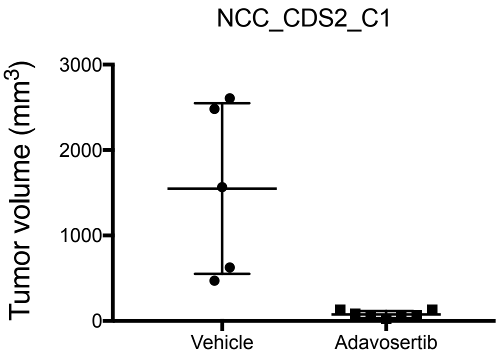

C

NCC_CDS1_X1_C1 (% change from baseline tumor volume)

Mouse. #	1	2	3	4	5	6	7	8
Vehicle	1115	1043	974	931	576	474		
Adavosertib	-28	-35	-37	-54	-66	-74	-81	-88

D

NCC_CDS2_C1 (% change from baseline tumor volume)

Mouse. #	1	2	3	4	5	6
Vehicle	1684	1205	770	681	494	
Adavosertib	-46	-51	-65	-66	-68	-69

Supplementary Figure 5. Adavosertib decreases CIC-DUX4 tumor volume compared to vehicle control. Comparative tumor xenograft size between adavosertib treated mice bearing NCC_CDS1_X1_C1 (A) or NCC_CDS2_C1 (B) compared to vehicle control. (C) Individual data points from figure 4C demonstrating the % change from baseline tumor volume in each NCC_CDS1_X1_C1 tumor bearing mouse. (D) Individual data points from figure 4C demonstrating the % change from baseline tumor volume in each NCC_CDS2_C1 tumor bearing mouse.

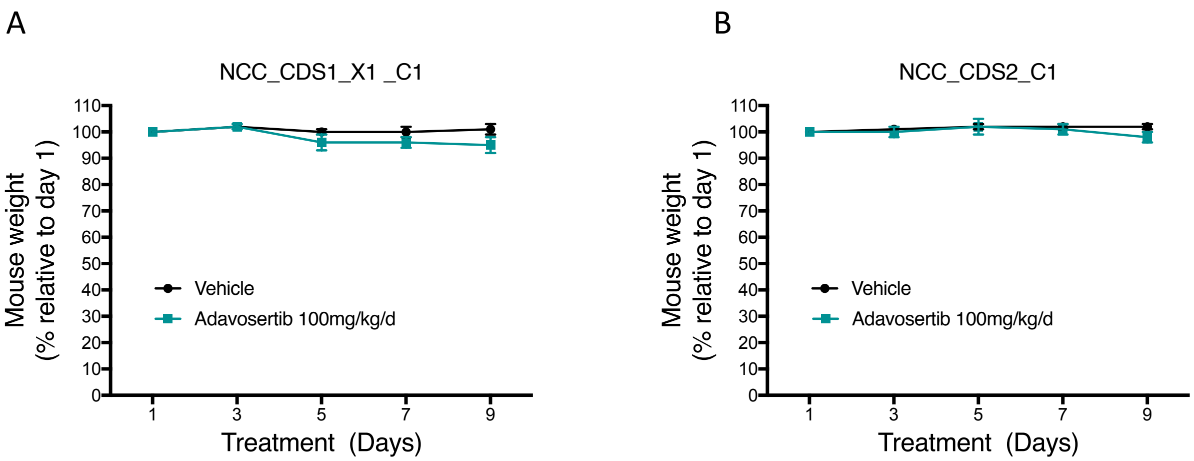

Supplementary Figure 6. Adavosertib is well tolerated in mice.

Serial weights of mice harboring NCC_CDS1_X1_C1 (A) and NCC_CDS2_C1 (B) xenografts treated with adavosertib or vehicle control.

Supplementary Table 1. 1426 differentially expressed genes in IB120 cells +/- CIC-DUX4 KD.
